# Multiple schwannoma of the seminal vesicle: A case report

**DOI:** 10.1097/MD.0000000000021603

**Published:** 2020-08-14

**Authors:** Yan Zhang, Jing Zhao, Pan Xu, Qi Qi

**Affiliations:** a Department of Medical Ultrasound, The First Affiliated Hospital of Nanchang University; b Department of Radiology, Jiangxi Maternal and Child Health Hospital, Nanchang, China.

**Keywords:** magnetic resonance imaging, multiple, schwannoma, seminal vesicle, ultrasonography

## Abstract

**Rationale:**

Schwannomas of the seminal vesicles are extremely rare, and only cases of single seminal vesicle schwannomas have been reported. Here, we report a case of multiple schwannoma of the seminal vesicle.

**Patient concerns:**

We report a rare case of multiple schwannoma of the seminal vesicle that occurred in a 48-year-old man during physical examination. Multiple mixed masses in the left region of the seminal vesicle were documented with transrectal ultrasonography and magnetic resonance imaging. The patient presented no clinical symptoms, no family history of the disease and no history of genetic disease.

**Diagnosis:**

Postoperative pathology revealed a diagnosis of seminal vesical schwannoma.

**Interventions:**

The patient underwent robotic-assisted laparoscopic surgery to remove the mass.

**Outcomes:**

The patient recovered rapidly and the length of hospitalization was 6 days after operation. At present, there is no recurrence in 10 month follow up.

**Lessons:**

Whether benign or malignant, single or multiple, schwannomas still need to be diagnosed by pathology because of the limitations of examination methods. Surgical resection is still the preferred treatment.

## 1 Introduction

Neurilemmoma, also called schwannoma, is usually a benign tumor with slow growth, a complete capsule and occasional malignant changes.^[1]^ This type of tumor often occurs in the head, neck, mediastinum, or retroperitoneum and rarely occurs in the urinary system, especially in the seminal vesicles. Only 10 cases of single seminal vesicle schwannomas have been reported.^[2–11]^ Multiple schwannomas are still rarer in the seminal vesicle, and they are only reported in the penis of the urinary system.^[12]^ Here, we report a rare case of multiple schwannoma of the seminal vesicle and its related clinical features.

## 2 Case report

**Figure 1:**
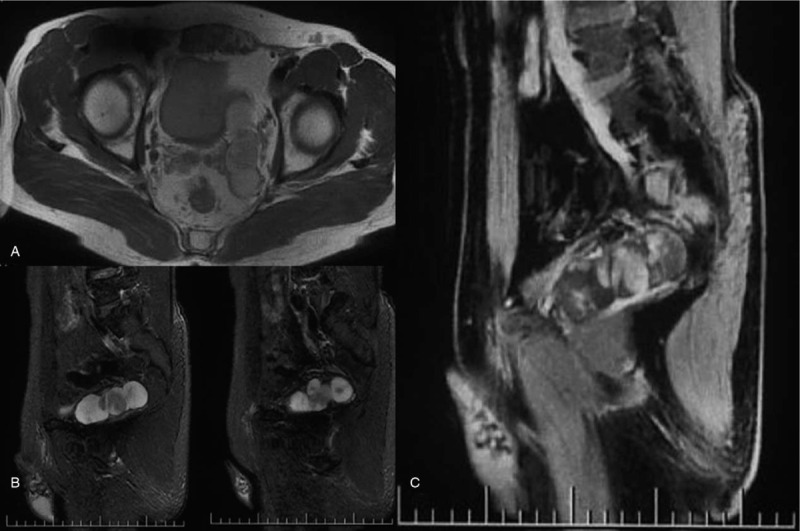
A: The lesion was dominated by long T1 and T2 signals, and the solid portion showed equal signal intensity; **Figure 1B:** the cystic components showed higher signal in the sagittal T2 lipid-pressing sequence; **Figure 1C:** the cyst wall and solid portion of the T1 lipid-pressing enhancement sequence showed progressive enhancement.

**Figure 2:**
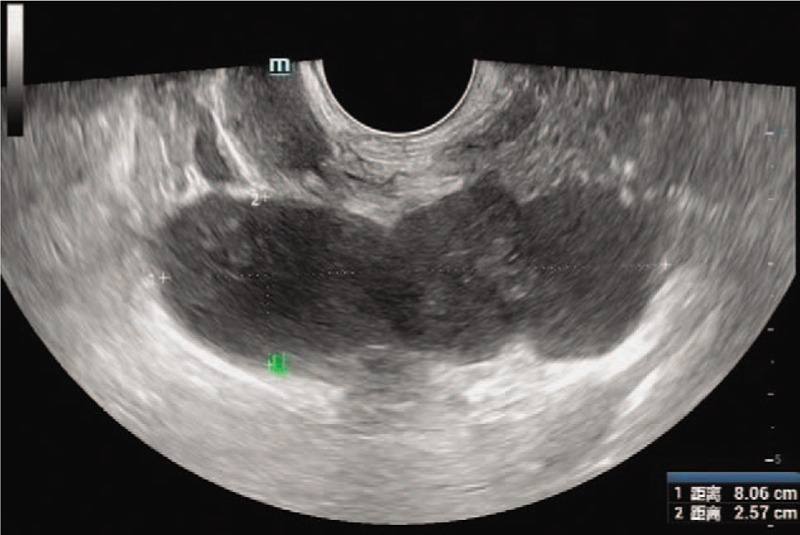
The left seminal vesicle was enlarged, and the inhomogeneous hypoechoic area was observed.

A 48-year-old married man who underwent magnetic resonance examination in a local hospital was found to have a mixed mass in the left seminal vesicle. In order to seek further treatment to our hospital, the patient denied any related clinical symptoms, family history of the disease, or history of genetic disease. Routine blood examination, coagulation function, prostate-specific antigen, and tumor markers were all within the normal ranges. We observed 4 independent but closely adjacent cystic-solid mixed masses in the left seminal vesicle through a scan provided by the patient (Fig. 1), the left margin of bladder was slightly compressed; the sizes were 3.5∗2.7 cm, 3.0∗2.4 cm, 2.7∗2.1 cm and 1.5∗1.3 cm, respectively. The lesion was dominated by long T1 and T2 signals, and the solid portion showed equal signal intensity, while the cystic components showed higher signal in the sagittal T2 lipid-pressing sequence. The cyst wall and solid portion of the T1 lipid-pressing enhancement sequence showed progressive enhancement. We observed that the edge of the whole lesion was smooth and clear. The left seminal vesicle was enlarged according to transrectal ultrasonography, with a visible range of 8.1∗2.6 cm in the inhomogeneous hypoechoic area (Fig. 2), and color Doppler showed a slight increase in blood flow signals. Robotic-assisted laparoscopic surgery was performed on July 31, 2018. During the operation, cystic and solid mixed tumors of the left seminal vesicle with a complete capsule and a clear demarcation from the surrounding tissues were observed. The operation lasted approximately 2 hours, and the estimated blood loss was 100 mL.

**Figure 3:**
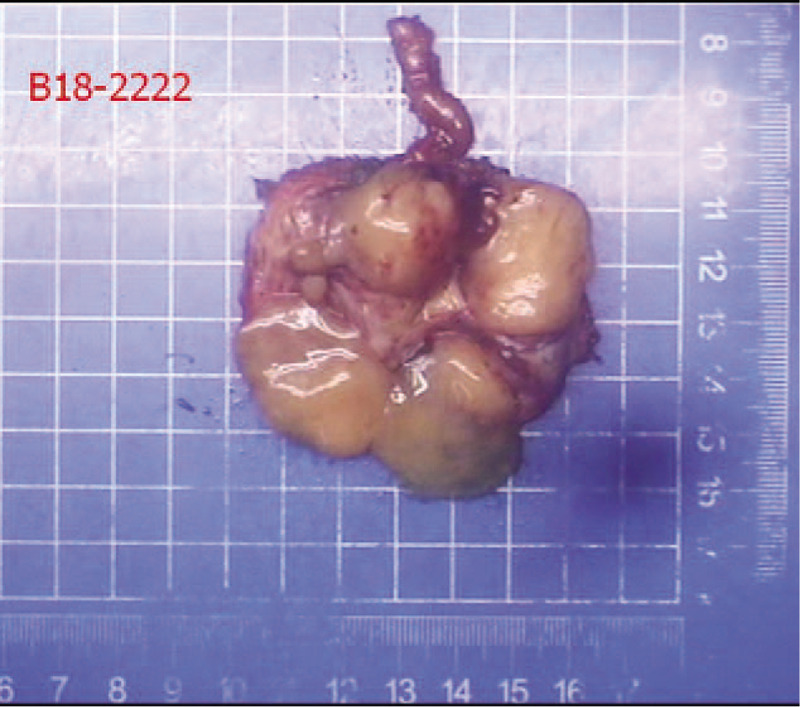
Shows a cystic lesion on the whole.

**Figure 4:**
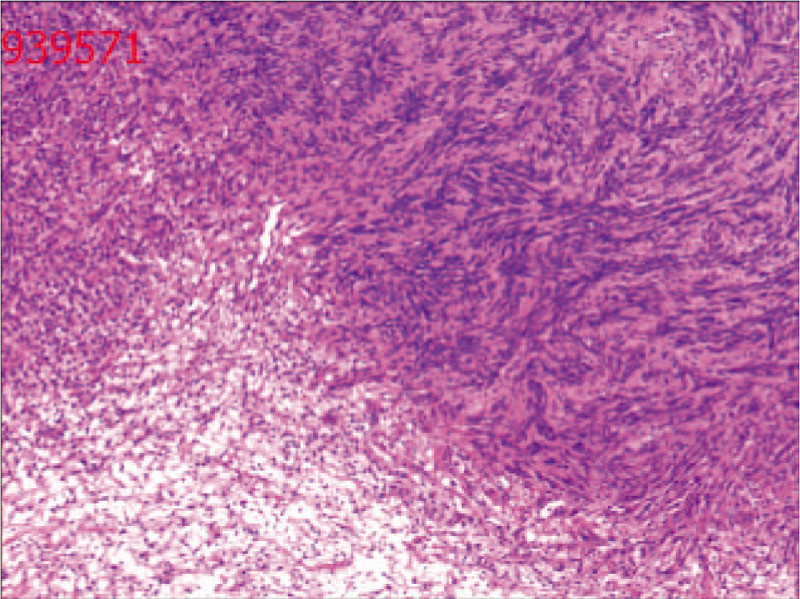
Microscopically, the tumor cells were spindle-shaped, bundled and focal palisade-shaped.

General appearance (Fig. 3): The section of tissue was cystic, and a gray-white solid area could be seen in the cyst. The solid area was described as nodular, soft and jelly-like, with a diameter of approximately 2.8 cm. Postoperative pathology revealed a diagnosis of seminal vesical schwannoma. Microscopically, the tumor cells were spindle-shaped, bundled and focal palisade-shaped (Fig. 4). The patient did not have any postoperative complications and was discharged safely on the 6th day after surgery. During the 10-month follow-up period, no clinical symptoms or signs of local recurrence were found.

## 3 Discussion

Schwannomas are relatively common benign tumors of peripheral nerves. Schwannomas can occur spontaneously or in the context of a familial tumor syndrome such as neurofibromatosis type 2 (NF2), schwannomatosis or Carney complex.^[13]^ At present, there were only 10 cases of single seminal vesicle schwannomas have been reported in the literature. Andrea et al^[7]^ reported a 2.6-cm single schwannoma of the seminal vesicle in a 43-year-old man with lower urinary tract symptoms. Arun et al^[9]^ reported a 13.5-cm single schwannoma of the seminal vesicle in a 50-year-old man who was found to have left abdominal pain that lasted for 1 month. Elmer-DeWitt et al^[10]^ reported that a 5.3-cm single schwannoma was found in the seminal vesicle adjacent to the rectum in a 62-year-old man with rectal pain, and this case was the first to report removal of the tumor by robotic surgery. Huang et al^[11]^ reported a 5.1-cm single schwannoma of the left seminal vesicle in a 55-year-old man after physical examination. According to these existing reports, schwannoma of the seminal vesicle usually does not cause clinical symptoms; however, when the surrounding tissues are compressed, there are some nonspecific symptoms, such as hemospermia, pain, irritation, obstructive lower urinary tract symptoms and infertility.^[3,14]^

Ultrasonography, computed tomography (CT) and magnetic resonance imaging (MRI) are the common imaging methods used to examination urologic neoplasms. Transrectal ultrasonography is the first-line method to detect urogenital tumors, and it can dynamically visualize the lesions.^[14]^ The reason why the number of lesions was not observed in this case was that the lesions had similar intervals or overlaps, and echoes were similar, and it is difficult to display the spatial relationship of the mass by two-dimensional ultrasound. Transrectal ultrasound could only be used to describe the general echoes of the lesions as well as the boundary and blood flow. However, because of its convenience and low cost, transrectal ultrasound can still be used as the preferred method for primary screening and follow-up after operation. CT and MRI can evaluate the nature of a mass and the degree of infiltration surround the mass, providing a basis for the choice of operation method. Neurilemmoma tumor cells generally consist of Antoni A and Antoni B regions as assessed by microscopy, and its imaging characteristics are different because of the different distribution of Antonia A and Antonib B in different tissues. Hideyuki et al^[15]^ by observing the imaging and pathological features of 162 cases of peripheral neurilemmoma, found that the area of Antoni A is situated in the central portion and the area of Antoni B is in the peripheral portion. On T2-weighted images, the area of Antoni B (in the peripheral portion) with edematous stroma shows relatively higher intensity than the area of Antoni A (in the central portion); on Gd-enhanced T1-weighted images, focal enhancement in the area of Antoni A with rich capillaries shows higher intensity than focal enhancement in the area of Antoni B. The author holds that the target sign should be defined as the characteristic central/peripheral biphasic pattern seen on T2-weighted images and Gd-enhanced T1-weighted images, MRI is the best way to connect macroscopic and microscopic findings. However, these characteristic imaging findings are not found in neurofibroma and other soft tissue tumors that are difficult to distinguish. We report a case of multiple seminal vesicle schwannoma, in which the cystic component (Antoni B region) showed higher signal in the sagittal T2 lipid-pressing sequence, and the cyst wall and solid portion (Antoni A region) of the T1 lipid-pressing enhancement sequence showed progressive enhancement. Because of more cystic degeneration, the biphasic pattern on MRI is not typical, which is difficult to diagnose. However, the imaging feature of the bipolar pattern of schwannoma on MRI provides a diagnostic method for similar cases in the future. In the left seminal vesicle of the patient, the boundary of the lesion was smooth and clear. Although the adjacent tissues were compressed, there was no sign of infiltration, suggesting a benign lesion. Benign schwannoma of the seminal vesicle should be differentiated from cystadenoma and low-grade epithelial-stromal tumors. Cystadenoma of the seminal vesicle is rare and can manifest as a septal multilocular neoplasm. The histological findings demonstrated that the tumors were composed of glands and cysts that were lined predominantly by cuboidal and columnar epithelium surrounded by fibrous stroma.^[16]^ Reikie et al^[17]^ proposed that the term seminal vesicle “mixed epithelial-stromal tumor” should be used to designate tumors of the seminal vesicle containing epithelial and stromal components. According to their criterion, cystadenoma of the seminal vesicle can be considered a low-grade mixed epithelial-stromal tumor.

For benign or malignant, single or multiple, schwannomas still need to be diagnosed by pathology because of the limitations of examination methods. Surgical resection is the first choice of treatment. In principle, the tumor should be thoroughly removed, and the surrounding organs, large vessels, and nerves should be maintained. There are various operative methods that can be chosen. The individual conditions of patients should be considered comprehensively, and the most suitable individual surgical scheme should be administered to patients. Traditional open surgery is time-consuming and involves intraoperative blood loss and trauma, and it easy to damage the surrounding organs such as the rectum with this method. Laparoscopy can reveal the deep seminal vesicles and surrounding tissues, and this procedure is relatively simple, safe and minimally invasive. Robot-assisted laparoscopic mass resection was used in this case. The patient was discharged on the sixth day after the operation and recovered quickly. The patient did not show any signs of recurrence at the 10-month follow-up. No further immunohistochemistry was performed in this case, continued vigilance is necessary for the possibility of recurrence, and this patient should be followed up for a long period of time.

## Acknowledgments

The authors would like to thank the patient and all members of the study team.

## Author contributions

**Conceptualization:** Yan Zhang, Jing Zhao, Pan Xu, Qi Qi.

**Data curation:** Yan Zhang, Qi Qi.

**Resources:** Pan Xu.

**Validation:** Pan Xu, Qi Qi.

**Writing – original draft:** Yan Zhang, Jing Zhao.

**Writing – review & editing:** Pan Xu.
